# Causes and trends of late diagnosis in Korean patients with hydroxychloroquine retinopathy

**DOI:** 10.3389/fmed.2023.1238226

**Published:** 2023-09-21

**Authors:** Seong Joon Ahn, Ji Hong Kim

**Affiliations:** Department of Ophthalmology, Hanyang University Hospital, Hanyang University College of Medicine, Seoul, Republic of Korea

**Keywords:** cause, hydroxychloroquine retinopathy, late diagnosis, referral, screening

## Abstract

**Introduction:**

Late diagnosis of hydroxychloroquine retinopathy remains a major concern, with the potential for irreversible visual impairment. This study aimed to investigate the causes of late diagnosis in a hospital-based cohort of Korean patients with hydroxychloroquine retinopathy and assess the trend of late diagnosis from 2015 to 2022.

**Methods:**

Thirty-eight patients with a late diagnosis (severe stage at diagnosis) among 94 patients with hydroxychloroquine retinopathy were included in the analysis. The causes of late diagnosis were categorized as referral-related, patient-related, and screening-related factors.

**Results:**

The most prevalent cause was no or late referral to ophthalmologists, contributing to a significant gap in timely identification. Patient-related causes included delayed monitoring visits despite scheduled appointments and early-onset disease. Screening-related causes encompassed an insufficient number of sensitive tests, leading to inadequate evidence for diagnosis, and missed or wrong diagnoses by screening physicians. The proportion of late diagnoses decreased over time, indicating improvements in overall screening and detection. The decreasing proportions of screening-related causes suggest advancements in screening practices and the use of multiple sensitive tests for screening.

**Discussion:**

Efforts to further reduce late diagnoses and improve screening and diagnostic processes are necessary. Our data emphasize the importance of timely referral to ophthalmologists for early detection and management.

## 1. Introduction

Hydroxychloroquine (HCQ) is widely prescribed for the treatment of various rheumatic and autoimmune diseases, including systemic lupus erythematosus and rheumatoid arthritis (1–3). However, its long-term use is associated with the risk of retinal toxicity, known as hydroxychloroquine retinopathy. Early detection and timely intervention are crucial for preventing irreversible visual impairment in patients undergoing hydroxychloroquine therapy (2, 4).

The American Academy of Ophthalmology (AAO) published guidelines in 2016 outlining the recommended practices for screening and monitoring patients undergoing HCQ treatment (2). These guidelines provide a framework for ophthalmologists and healthcare providers to ensure the adequate surveillance and early diagnosis of hydroxychloroquine retinopathy. However, despite the availability of these guidelines, the late diagnosis of hydroxychloroquine retinopathy continues to be a concern, leading to significant visual impairment in affected individuals (2, 5, 6).

The late diagnosis of hydroxychloroquine retinopathy in Asian patients could also be attributed to the unique pericentral pattern of retinopathy observed in this population (5–9). Studies have shown that Asian individuals may be more prone to developing a pericentral pattern of retinopathy characterized by the initial involvement of the retina around the major arcade (5, 6, 8, 9). This pattern can be more challenging to detect in its early stages using conventional screening modalities that primarily focus on the macula (5, 10). Consequently, delays in diagnosis may occur because the macular area is covered by conventional optical coherence tomography (OCT) scans, which are typically spared in the early stages of pericentral retinopathy, and gradually become affected with disease progression (5, 10). However, a recent study on nationwide practice patterns in South Korea revealed that the proportion of patients receiving appropriate baseline and monitoring examinations was less than one-third, indicating that unscreened long-term user can be an important cause of late diagnosis in Asian patients (11). However, the causes of late diagnoses have not been specifically investigated in Asian patients.

Accordingly, this study aimed to investigate the causes of late diagnosis in a hospital-based cohort of Korean patients with hydroxychloroquine retinopathy by focusing on referral-, patient-, and screening-related causes. Furthermore, we analyzed the temporal pattern of late diagnosis among patients diagnosed with hydroxychloroquine retinopathy in the cohort, aiming to identify any recent changes that may have occurred over time.

## 2. Methods

### 2.1. Participants

Ninety-four patients diagnosed with hydroxychloroquine retinopathy at Hanyang University Hospital between January 2005 and May 2023 were included in this study. They were diagnosed with hydroxychloroquine retinopathy based on at least two of the 4 tests including OCT, Humphrey visual field test (VF), fundus autofluorescence, and multifocal electroretinography (mostly OCT evidence confirming VF abnormality). The study was conducted in accordance with the principles of the Declaration of Helsinki and was approved by the Institutional Review Board (IRB) of Hanyang University Hospital. The requirement for informed consent was waived by the IRB board owing to the retrospective nature of the study.

### 2.2. Evaluations and data collection

All screened patients underwent a comprehensive ophthalmic examination, which included measurements of best-corrected visual acuity, slit-lamp examination, and fundus examination. OCT imaging was performed using Stratus OCT (Carl Zeiss Meditec, Dublin, CA, United States), 3D-OCT 2000 (Topcon Inc., Tokyo, Japan), or DRI-OCT (Topcon Inc., Tokyo, Japan) and all the images were reviewed for our analyses. Fundus autofluorescence (FAF) was performed using an F-10 confocal scanning laser ophthalmoscope (cSLO; Nidek, Gamagori, Japan) or ultra-widefield FAF (Optos 200T×; Optos PLC, Dunfermline, United Kingdom). All patients received Humphrey automated perimetry (Humphrey Field Analyzer II or III; Carl Zeiss Meditec, Inc.) using the 30-2 and/or 10-2 protocol for diagnosis of hydroxychloroquine retinopathy. mfERG (Diagnosys LLC, Lowell, MA, United States) was performed in selected cases requiring additional objective evidence of retinal toxicity according to the guidelines of the International Society for Clinical Electrophysiology of Vision (ISCEV) (12).

Patient records from multiple ophthalmology centers were reviewed to extract relevant data pertaining to referrals by prescribing physicians, patient-related factors, and screening practices. Data collected from electronic medical records included baseline demographic factors, such as age, sex, and body weight, as well as past medical history. Information regarding hydroxychloroquine use, including the start date, daily dose, and duration of use, was recorded. Furthermore, all information on referrals for retinopathy screening was collected using referral letters and medical records written by prescribing physicians or ophthalmologists. The purpose of the visit, referral by prescribing physicians or patients’ visual complaints, were retrieved from the medical records by ophthalmologists. This information was confirmed using referral letters (from outside hospitals and our institution) or medical records by prescribing physicians.

Screening details, such as the timing and modalities used for the four tests recommended in the 2016 AAO guidelines as well as any ancillary tests performed, were also documented. The scheduled follow-up visits and dates of the actual follow-up visits were recorded. Specifically, the use of wide-scan OCT (scan length of 9 mm or 30° or larger for OCT), wider automated visual fields (24-2 or 30-2), and wide-field FAF (retinal anatomic features beyond the posterior pole in all four quadrants) was evaluated (2, 13, 14).

### 2.3. Assessment of causes of late diagnosis

**Figure 1:**
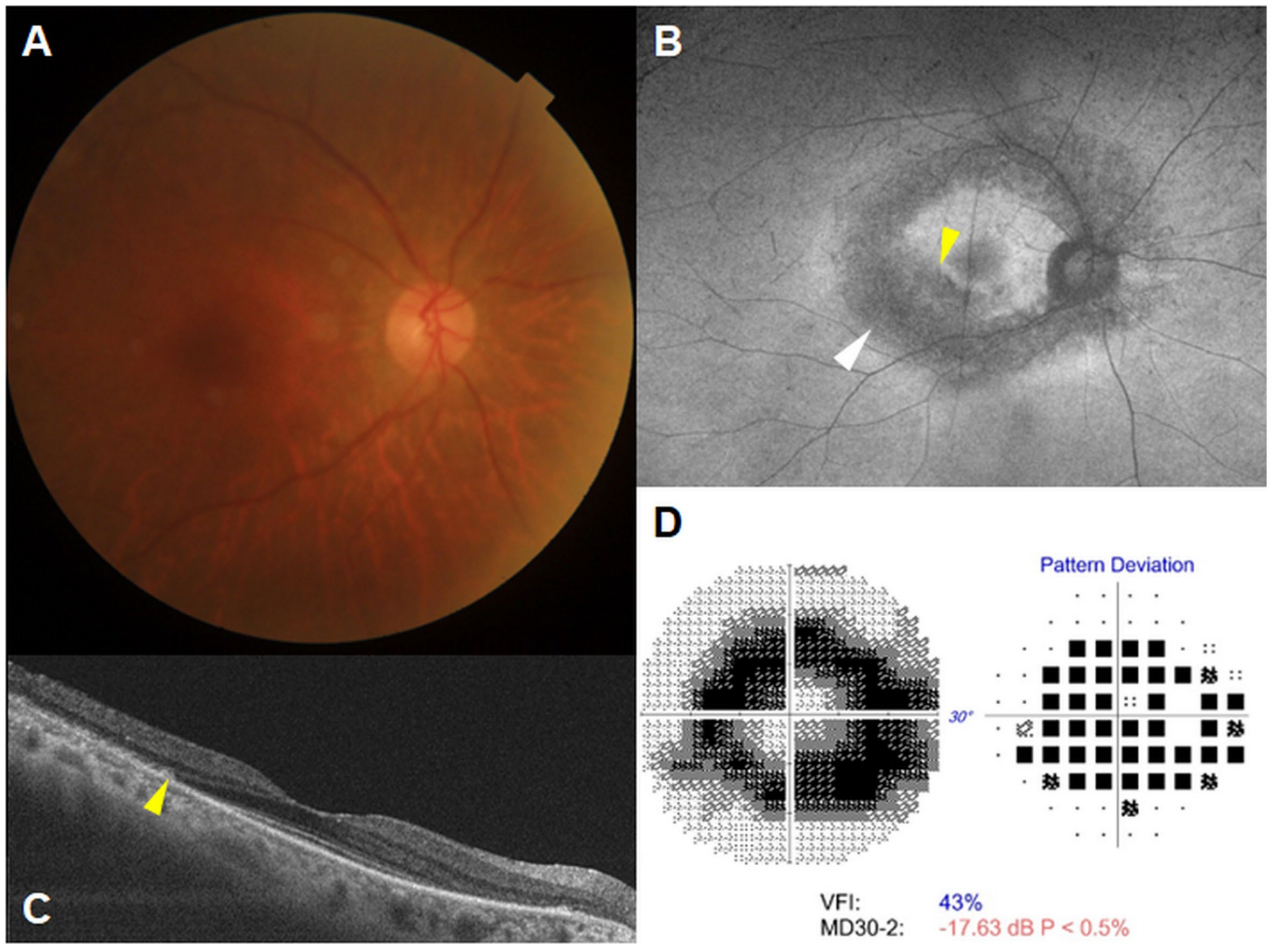
A representative case illustrating late diagnosis of hydroxychloroquine retinopathy in a 66 year-old female who had been on hydroxychloroquine treatment for 20 years, without prior referral for retinopathy screening from prescribing physicians. Fundus photograph **(A)** and fundus autofluorescence (FAF) **(B)** show hypoautofluorescence in the parafoveal (indicated by the yellow arrowhead) and pericentral areas (indicated by the white arrowhead) on FAF. Optical coherence tomography **(C)** and Humphrey 30-2 test **(D)** reveal parafoveal and pericentral photoreceptor loss with retinal pigment epithelial changes (indicated by the yellow arrowhead) and a ring scotoma, respectively. MD, mean deviation; VFI, visual field index.

A late diagnosis was defined as a diagnosis of hydroxychloroquine retinopathy at a severe stage (hypoautofluorescence on FAF and/or thinned/attenuated RPE on OCT as illustrated in Figure 1) at the time of diagnosis (6, 15). This was based on the previous findings that eyes with severe stages showed progressive changes despite drug cessation, whereas those with early or moderate stages revealed stable changes after the cessation or even partial recovery (5, 16). In patients with late diagnosis, patient records were reviewed by two independent reviewers (SA and JK) to identify the causes of late diagnosis, related to referral for retinopathy screening, and patient- and screening-related causes in those with late diagnosis. Any disparity in the judgment was resolved through discussion until consensus was reached.

More specifically, if patients did not undergo timely ophthalmic examination (retinopathy screening), referral- and patient-related causes were investigated. For all annual screening examinations, we evaluated whether screening was performed appropriately according to the current guidelines (2). Patients were determined to have screening-related causes if appropriate annual screening was not performed using at least two of the four modalities recommended by the AAO; a wider test was not used for screening, the diagnosis was missed or wrong, or the next appointment, either annually or earlier, was not made for regular monitoring.

The evaluation of structural and functional defects was conducted for each cause of late diagnosis using OCT and VF. For each cause, the structural defects were measured using the extent of hypo-autofluorescent lesion (in hours) and the distance from the fovea to the outer retinal defects (in μm), providing information on the circumferential and centripetal progression of retinal damage and its threat to the fovea. In addition, functional parameters, namely mean deviation (MD) and visual field index (VFI) from the Humphrey field analyzer 30-2 test, were also analyzed for the eyes categorized under each cause.

### 2.4. Data analysis

Data on the causes of late diagnoses were tabulated and categorized based on referral-, patient-, and screening-related factors. Descriptive statistics were used to summarize the demographic and clinical characteristics of the study population. Comparative analyses were conducted between pre- and post-guideline implementation periods to assess the impact of the guidelines on the rates and causes of late diagnoses. Statistical methods such as chi-square tests or *t*-tests were used to identify significant differences. All statistical analyses were performed using SPSS software (version 27.0; IBM Corp.), and statistical significance was set at *p* < 0.05. *p*-values ranging from 0.05 to 0.1 were considered marginally significant.

## 3. Results

### 3.1. Demographic and clinical characteristics

Thirty-eight patients with a late diagnosis were included in the analysis. The demographic and clinical characteristics of the patients are shown in Table 1. The mean age at the time of diagnosis was 62.2 ± 11.7 years, with a range of 29 to 81 years. Rheumatoid arthritis (RA) was the most common indication for hydroxychloroquine therapy among the patients in this cohort. Approximately 60% of the included patients exhibited retinal damage restricted to the pericentral area, whereas the remaining patients showed involvement of the parafoveal region. The mean duration of hydroxychloroquine use was 210.9 ± 90.2 months, and the average daily dose adjusted to real body weight was 5.0 ± 1.7 mg/kg.

**Table 1:** Demographic and clinical characteristics of the patients (*n* = 38).

Characteristics	Values
Sex, female (%)	37 (97.4%)
Age, years	62.2 ± 11.7
*Diagnosis (%)*	
SLE: RA: others	15 (39.5%): 20 (52.6%): 3 (7.9%)
*Pattern of retinopathy, eyes (%)*	
Parafoveal: pericentral: mixed	9 (11.8%): 19 (25%): 48 (63.2%)
Mean daily dose, mg	265.3 ± 87.7
Mean daily dose/real body weight, mg/kg	5.0 ± 1.7
Mean duration of hydroxychloroquine use, months	210.9 ± 90.2
Mean cumulative dose, g	1678.7 ± 887.2

SLE, systemic lupus erythematosus; RA, rheumatoid arthritis.

### 3.2. Causes of late diagnosis

The potential causes of late diagnosis in hydroxychloroquine retinopathy were categorized into different groups based on referral-, patient-, and screening-related factors. The causes and percentages of each item are summarized in Table 2.

**Table 2:** Potential causes of late diagnosis in our (all Korean) patients with hydroxychloroquine retinopathy.

Causes of late diagnosis	*N* (%)
*Referral-related causes*	
Absence of referral to an ophthalmologist for retinopathy screening	5 (13.2%)
Late referral to an ophthalmologist for retinopathy screening	22 (57.9%)
*Patient-related causes*	
Patient non-adherence to recommended screening appointments	6 (15.8%)
Early-onset disease on screenings performed as per the current guideline (screening appropriately performed after timely referral)	1 (2.6%)
*Screening-related causes*	
Monitoring performed using insensitive tests	8 (21.1%)
Appropriate annual screening not performed using at least two of the four modalities recommended by the AAO	8 (21.1%)
Wider test not used for screening	3 (7.9%)
Missed (diagnosis of no retinopathy despite evidences of toxicity present) or wrong diagnosis (diagnosis with other diseases such as glaucoma) by screening physicians	7 (18.4%)
No appointment made for the next (annual or earlier) screening	2 (5.3%)

#### 3.2.1. Referral-related causes

The prevalent cause of late diagnosis was lack of initial timely referral to ophthalmologists, with 27 patients (71.1%) not being referred (*n* = 5) or being referred late (*n* = 22) for retinopathy screening. This indicates a significant gap in the timely identification of retinopathy in these patients.

#### 3.2.2. Patient-related causes

Patients’ monitoring visits were delayed despite scheduled appointments in six patients (15.8%), contributing to the delay in diagnosis. One patient (2.9%) exhibited early onset disease, which may have led to delayed recognition of retinal toxicity, although appropriate screening was performed after timely referral by the prescribing physicians.

#### 3.2.3. Screening-related causes

Among the screening-related causes, the lack of sensitive tests was a prominent factor (21.1%), particularly monitoring which had been performed before the availability of spectral-domain optical coherence tomography (SD-OCT). Additionally, 8 patients (21.1%) had an insufficient number of tests performed, resulting in inadequate evidence for the diagnosis of retinopathy in previous screening examinations. Three patients (7.9%) did not undergo any wider OCT, FAF, or VF test for screening examinations performed before diagnosis. Missed or incorrect diagnoses by screening physicians despite evidences of retinal toxicity accounted for seven patients (18.4%), leading to later diagnosis. These patients had been misdiagnosed with retinitis pigmentosa, macular diseases such as age-related macular degeneration and resolved central serous chorioretinopathy, or glaucoma, or their retinopathy was missed in the previous screening, indicating potential errors or oversights in the screening process.

### 3.3. Structure and functional defects for each cause of late diagnosis

Table 3 presents the extent of retinal damage observed on optical coherence tomography (OCT) and the corresponding visual field indices obtained from standard (Humphrey) automated perimetry for various causes of late diagnosis. Under referral-related causes, the absence of referral to an ophthalmologist for retinopathy screening resulted in an average extent of hypo-autofluorescent lesion of 11.3 ± 1.6 h, a distance of 787.0 ± 766.0 μm from the foveal center to the outer retinal defects, an MD of −22.6 ± 9.0, and a VFI of 34.2 ± 31.1. Late referral to an ophthalmologist for retinopathy screening showed similar measurements with slightly less damage compared to the absence of referral. However, patient-related and screening-related causes showed lesser damage than referral-related causes with the less average extent of hypo-autofluorescent lesion, greater distance from the foveal center to the outer retinal defects, and better MD and VFI.

**Table 3:** Extent of retinal damage on optical coherence tomography and visual field indices on automated perimetry for each cause of late diagnosis.

Causes	Structure		Function (perimetry)	
	Extent of hypo-autofluorescent lesion (hours)	Distance from the fovea to the outer retinal defects, μm	Mean deviation (MD)	Visual field index (VFI)
*Referral-related causes*				
Absence of referral to an ophthalmologist for retinopathy screening	11.3 ± 1.6	787.0 ± 766.0	−22.6 ± 9.0	34.2 ± 31.1
Late referral to an ophthalmologist for retinopathy screening	9.1 ± 4.1	721.4 ± 723.7	−18.1 ± 7.8	42.6 ± 26.0
*Patient-related causes*				
Patient non-adherence to recommended screening appointments	7.4 ± 3.4	1301.4 ± 1163.3	−11.4 ± 10.4	67.0 ± 34.5
Early-onset disease on screenings performed as per the current guideline (screening appropriately performed after timely referral)	9.5 ± 0.7	1463.0 ± 106.1	−1.92 ± 0.24	96.0 ± 1.4
*Screening-related causes*				
Monitoring performed using insensitive tests	8.9 ± 3.3	1460.8 ± 1124.9	−15.8 ± 7.9	56.3 ± 27.4
Appropriate annual screening not performed using at least two of the four modalities recommended by the AAO	8.9 ± 3.3	1327.9 ± 1227.9	−16.7 ± 9.2	52.3 ± 33.0
Wider test not used for screening	7.7 ± 4.0	725.5 ± 975.5	−12.4 ± 4.1	66.0 ± 14.0
Missed (diagnosis of no retinopathy despite evidences of toxicity present) or wrong diagnosis (diagnosis with other diseases such as glaucoma) by screening physicians	10.4 ± 2.4	1049.9 ± 1008.5	−20.1 ± 6.3	40.6 ± 24.6
No appointment made for the next (annual or earlier) screening	5.8 ± 4.2	2181.0 ± 1287.3	−9.7 ± 5.1	77.0 ± 18.2

^*^Humphrey field analyzer 30-2 test was used.

### 3.4. Trend of late diagnosis

**Figure 2:**
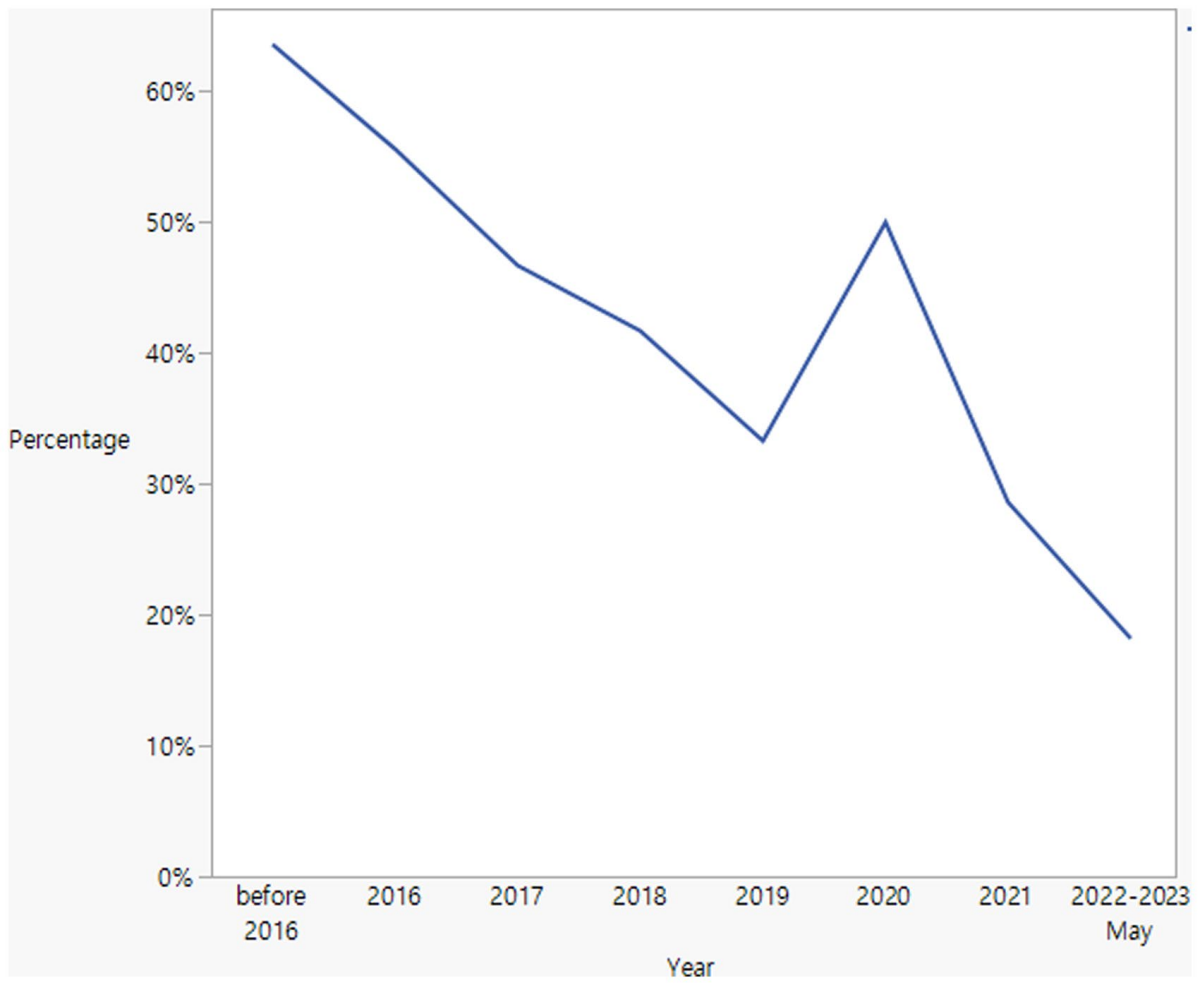
Annual trend of late diagnosis among patients diagnosed with hydroxychloroquine retinopathy.

**Figure 3:**
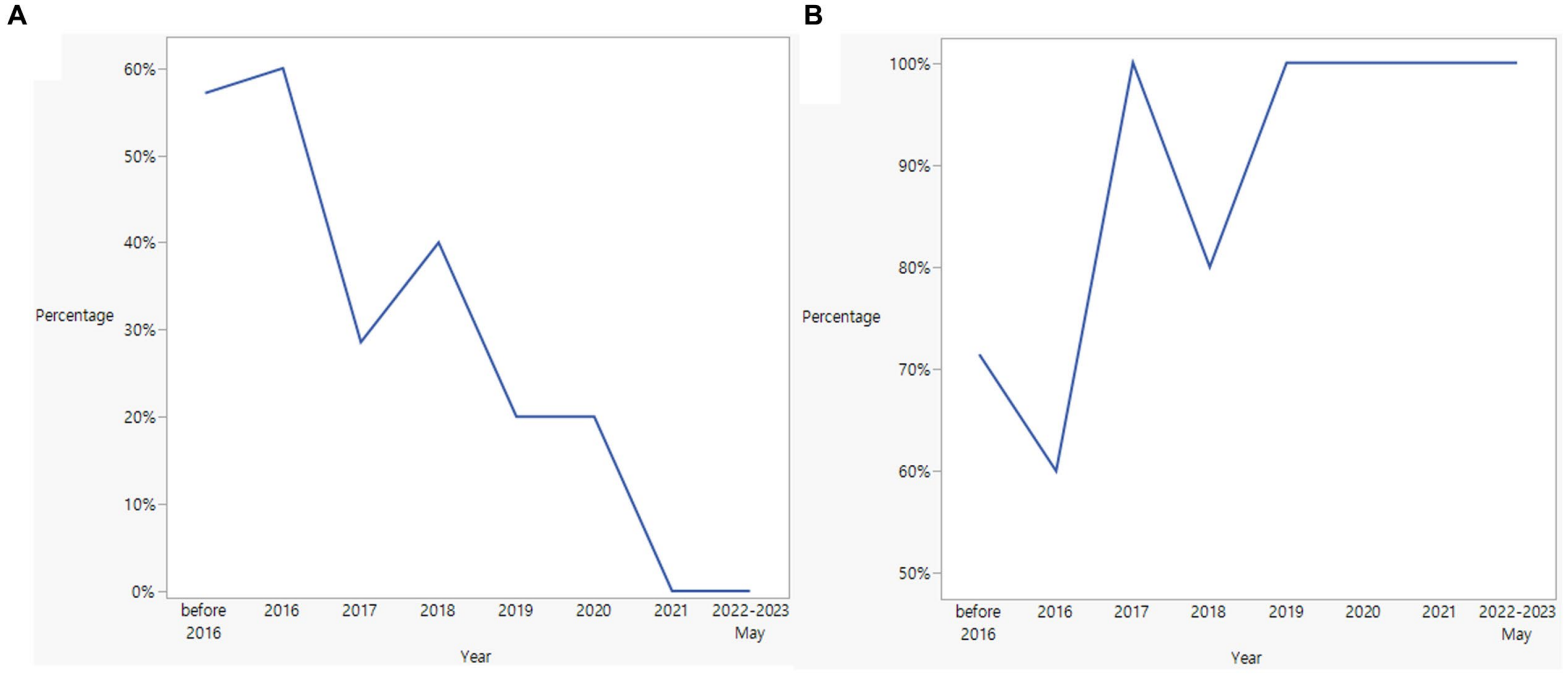
Trends of screening-related **(A)** and other causes **(B)** for late diagnosis in Asian patients with hydroxychloroquine retinopathy.

Figure 2 illustrates the proportion of patients diagnosed with hydroxychloroquine retinopathy each year who received a late diagnosis. In general, the percentage of late diagnoses exhibited a gradual decline from the period before 2016 (63.6%) to the year 2022 (18.2%). Figure 3 depicts the yearly trend in the distribution of causes for late diagnosis, differentiating between screening-related causes and others. Notably, the proportion of patients with screening-related causes has notably decreased in recent years.

Between before and after Year 2016, the year the current AAO guidelines were published, there were marginally significant differences in the percentage of patients with a late diagnosis (63.6% vs. 37.1%, *p* = 0.096). Screening-related causes were also different between patients diagnosed before and after 2016 (57.1% and 23.1%, respectively); however, the difference was not statistically significant (*p* = 0.161).

## 4. Discussion

The results of this study shed light on the causes and trends associated with the late diagnosis of hydroxychloroquine retinopathy, which remains a significant concern for this condition. Our study utilized a retrospective analysis of patient records in a tertiary hospital to investigate and categorize several potential causes of late diagnosis in Korean patients with hydroxychloroquine retinopathy. Understanding the factors contributing to a delayed diagnosis is crucial for improving the timely identification and management of this condition, thereby minimizing potential vision loss in patients taking the drug.

Analysis of the included patients revealed several categories of causes for late diagnosis, namely, referral-, patient-, and screening-related factors. The most prevalent, important cause was the lack of timely referral to ophthalmologists. No or late referral was identified as the most damaging cause, as the included eyes exhibited the greatest extent of photoreceptor defects threatening the fovea and also displayed the worst perimetric function. This finding highlights a significant gap in appropriate screening as per the current guidelines and the timely identification of retinal toxicity, as a large proportion of patients were either not referred or were referred late for retinopathy screening. Efforts should be made to enhance awareness among prescribing physicians such as rheumatologists and dermatologists regarding the potential retinal toxicity associated with hydroxychloroquine use, ensuring that patients are promptly referred for ophthalmological evaluation after its use (13).

Patient-related causes were also identified as contributors to late diagnoses, with delayed monitoring visits despite scheduled appointments being a notable factor. This emphasizes the importance of patient compliance and adherence to follow-up appointments, as regular monitoring is crucial for early detection of retinal changes associated with hydroxychloroquine use. Educating patients about the significance of these visits, namely the need for regular screening and the potential consequences of delayed monitoring, could help improve patient engagement and reduce delays in diagnosis.

Another category identified in this study was screening-related causes. The insufficient availability of sensitive tests such as SD-OCT has been found to affect a significant proportion of patients. SD-OCT is a valuable tool for detecting retinal changes in hydroxychloroquine retinopathy (7), and its unavailability before 2010 at our institution may remarkably hinder the timely identification of the condition. Efforts should be made to ensure the availability and utilization of such advanced imaging techniques in screening programs (7), particularly for patients at high risk of retinopathy (e.g., long-term hydroxychloroquine users and daily dose/body weight >5 mg/kg) (2).

Furthermore, inadequate evidence for diagnosis and missed or incorrect diagnoses by screening physicians were identified as screening-related causes that contributed to late diagnosis. These findings emphasize the significance of screening physicians’ awareness and expertise regarding this condition. Although comprehensive and appropriate screening are performed in hydroxychloroquine users, missed or incorrect diagnoses may lead to continued therapy and significant vision loss due to progression of retinal toxicity. Therefore, appropriate training and updates are required for screening physicians to ensure the accurate interpretation of screening results and early detection of retinopathy.

Analysis of trends in late diagnoses over the years provides valuable insights into the progress made in diagnosing hydroxychloroquine retinopathy. The decreasing proportions of late diagnoses observed from 2015 to 2022 indicate improvements in the overall detection or referral for retinopathy screening. Notably, the proportion of patients with screening-related causes decreased over time, suggesting advancements in screening practices and the availability of sensitive tests, such as SD-OCT. This trend indicates a positive shift in screening for retinal toxicity associated with hydroxychloroquine use. National guidelines, such as the 2016 AAO recommendations, might have played a significant role in this shift (2). However, it is essential to continue efforts aimed at further reducing late diagnoses and improving the overall screening and diagnostic processes for retinopathy. This may involve targeted interventions such as educational initiatives for healthcare professionals and patient education programs emphasizing the importance of regular monitoring and ensuring the availability and utilization of advanced imaging technologies in screening practices.

Unfortunately, specific interventions or therapies for eyes with a late diagnosis of hydroxychloroquine retinopathy are currently lacking, aside from drug cessation. The pathogenic mechanism underlying retinal toxicity has not been fully elucidated, which may partly account for the absence of targeted treatments to reverse retinal damage. Discontinuing the use of the drug remains the primary management option for patients with hydroxychloroquine retinopathy, as it has demonstrated evidence of reducing disease progression by minimizing further drug-induced damage. Although dose lowering could be considered as an interventional strategy, there is insufficient evidence supporting its efficacy at present. Considering that previous studies have shown continuous progression even after drug cessation in severe stages at diagnosis, complete drug cessation may be more favorable than dose lowering for patients with late diagnosis, given the risk of significant vision loss in the patients. To address this unmet need, further research is warranted to explore and validate potential therapeutic approaches beyond drug cessation for managing late-stage hydroxychloroquine retinopathy.

This study has some limitations. The sample size was relatively small, which may limit conclusions regarding the causes of late diagnoses in Korean populations. Future studies should utilize a prospective design and include a larger number of patients, following careful sample size calculation. Furthermore, the study population may not represent the general population with hydroxychloroquine retinopathy, limiting the external validity of the findings. Moreover, it is important to note that “late stage disease” does not always equate to “late diagnosis,” although previous studies have often used the term to refer to severe disease at the time of diagnosis. However, there is currently no consensus on the exact definition of this term, highlighting the need for standardization or a consensus definition in future studies. Additionally, the study focused on the causes and trends of late diagnosis without exploring potential interventions or strategies to address these causes. Further research is warranted to investigate the impact of specific interventions and assess their effectiveness in reducing late diagnosis of hydroxychloroquine retinopathy. Finally, while it was not feasible to estimate the exact elapsed time of late diagnosis for the different causes in this study, determining the relative delay for each cause holds clinical significance in identifying the most detrimental cause concerning the elapsed time.

In conclusion, this study highlights the causes and trends of late diagnosis of hydroxychloroquine retinopathy. These findings emphasize the need for improved referral practices, enhanced patient compliance with monitoring visits, and the availability and utilization of sensitive screening tests. The observed decrease in the proportion of late diagnoses over time suggests progress in the screening of hydroxychloroquine retinopathy. Continued efforts to optimize screening protocols and educate healthcare professionals and patients regarding toxicity screening are essential to reduce late diagnoses and minimize visual loss due to the toxicity.

## Data availability statement

The datasets presented in this article are not readily available because the data used in this study are not publicly available due to privacy and ethical considerations. Requests to access the datasets should be directed to ahnsj81@gmail.com.

## Ethics statement

The studies involving humans were approved by the requirement for informed consent was waived by the IRB of Hanyang University Hospital. The studies were conducted in accordance with the local legislation and institutional requirements. The ethics committee/institutional review board waived the requirement of written informed consent for participation from the participants or the participants’ legal guardians/next of kin because owing to the retrospective nature of the study and deidentified data.

## Author contributions

SA: conception, design, and writing. SA and JK: analysis, data collection, interpretation, obtain funding, and overall responsibility. All authors contributed to the article and approved the submitted version.

## Funding

This work was supported by the National Research Foundation of Korea Grant funded by the Korean Government MSIT (NRF-2021R1G1A1013360).

## Conflict of interest

The authors declare that the research was conducted in the absence of any commercial or financial relationships that could be construed as a potential conflict of interest.

## Publisher’s note

All claims expressed in this article are solely those of the authors and do not necessarily represent those of their affiliated organizations, or those of the publisher, the editors and the reviewers. Any product that may be evaluated in this article, or claim that may be made by its manufacturer, is not guaranteed or endorsed by the publisher.
